# Calcitonin-Secreting Neuroendocrine Carcinoma of Larynx with Metastasis to Thyroid

**DOI:** 10.1155/2015/606389

**Published:** 2015-09-28

**Authors:** Lauren LaBryer, Ravindranauth Sawh, Colby McLaurin, R. Hal Scofield

**Affiliations:** ^1^Division of Endocrinology, Department of Internal Medicine, University of Oklahoma Health Sciences Center, Oklahoma City, OK 73104, USA; ^2^Oklahoma City VA Health Care Systems, Oklahoma City, OK 73104, USA; ^3^Department of Pathology, University of Oklahoma Health Sciences Center, Oklahoma City, OK 73104, USA; ^4^Department of Otolaryngology, University of Oklahoma Health Sciences Center, Oklahoma City, OK 73104, USA; ^5^Oklahoma Medical Research Foundation, Oklahoma City, OK 73104, USA

## Abstract

Primary neuroendocrine tumors of the larynx are rare, with moderately differentiated neuroendocrine carcinoma (MDNC) being the most frequent histologic type. We report a MDNC in a 57-year-old gentleman with an enlarging right-sided neck mass. Flexible fiberoptic exam revealed a right arytenoid lesion. Histology from excisional biopsy was concerning for medullary thyroid carcinoma (MTC) versus NET of the larynx. Immunohistochemistry was diffusely positive for calcitonin and CEA and focally positive for TTF-1. Serum calcitonin was elevated. Thyroid ultrasound was unremarkable. The patient underwent laryngectomy, thyroidectomy, and neck dissection. Pathology showed neuroendocrine carcinoma of right arytenoid with positive cervical lymph nodes. A 4 mm deposit of NET was present in right thyroid with adjacent intravascular tumor consistent with thyroidal metastasis from a primary laryngeal NET (MDNC). MDNC and MTC can be microscopically indistinguishable. Both tumors can stain positively for calcitonin and CEA. TTF-1 staining has been useful to help distinguish these tumors as it is strongly and diffusely positive in MTC, but usually negative (or only focally positive) in MDNC. We report the fourth case of primary neuroendocrine carcinoma of the larynx associated with elevated serum calcitonin level and the first such case associated with metastasis to the thyroid.

## 1. Introduction

Neuroendocrine tumors of the larynx are rare, accounting for ~0.6% of laryngeal neoplasms [1]. Four types of neuroendocrine tumors of the larynx have been identified by the WHO: well-differentiated neuroendocrine carcinoma (typical carcinoid), moderately differentiated neuroendocrine carcinoma (atypical carcinoid), poorly differentiated neuroendocrine carcinoma (small cell carcinoma, neuroendocrine type), and paraganglioma [2]. Moderately differentiated neuroendocrine carcinoma (MDNC) is the most frequent type of all neuroendocrine tumors of the larynx [2, 3].

MDNC of the larynx and medullary thyroid carcinoma (MTC) demonstrate similar morphological features and can be microscopically indistinguishable, particularly when presenting as metastasis [4]. Both tumors stain positively for calcitonin and CEA. TTF-1 staining has been useful to help distinguish these tumors as it is strongly and diffusely positive in medullary thyroid carcinoma, but usually negative (or only focally positive) in MDNC [2, 4]. To the best of our knowledge, only 3 cases of neuroendocrine carcinoma of the larynx with elevated serum calcitonin have been reported [5–7]. We report the fourth case of primary calcitonin-producing neuroendocrine tumor of the larynx. There are <20 cases of neuroendocrine tumors metastasizing to the thyroid [8–10]. This is the first case reported of a neuroendocrine tumor of larynx with suspected metastasis to the thyroid.

## 2. Case Presentation

A 57-year-old gentleman presented with 1-year history of an enlarging right-sided neck mass. The patient noted significant pain/tenderness around the mass with associated right-sided otalgia, odynophagia, and hoarseness. He was a former smoker and alcoholic with no other significant past medical history. There was no family history of cancer or endocrinopathy. Physical exam was remarkable for a 2.5 cm × 1.5 cm mass palpable in the right side of his neck. Flexible fiberoptic exam of the larynx showed a right medial arytenoid lesion of approximately 1 cm in size, mucosally covered with central ulceration. The patient underwent FNA of the palpable right neck mass. Initial pathology was concerning for metastatic carcinoma, favoring poorly differentiated adenocarcinoma of likely primary lung origin. Both PET scan and CT thorax failed to reveal significant lung pathology but rather redemonstrated the laryngeal lesion. The patient then underwent microlaryngoscopy with excisional biopsy of the right arytenoid mass en bloc with the superior aspect of the arytenoid cartilage. Immunohistochemistry was diffusely positive for calcitonin, polyclonal CEA, synaptophysin, chromogranin, and cytokeratin and focally positive for TTF-1. Pathology was concerning for medullary thyroid carcinoma versus neuroendocrine tumor of the larynx. Serum calcitonin was elevated at 157 pg/mL (ref 0–8 pg/mL). Serum CEA was normal. Thyroid ultrasound revealed no abnormalities of the thyroid. Ki-67 staining was 15%, consistent with a moderately differentiated neuroendocrine carcinoma. The case was discussed at our head and neck tumor board with recommendations for total laryngectomy and bilateral neck dissection given the diagnosis of MDNC with evidence of regional lymph node metastasis but no distant metastasis on PET scan. Total thyroidectomy was also recommended given the remaining question on pathology of MDNC versus MTC. The patient subsequently underwent total laryngectomy, bilateral neck dissection, and total thyroidectomy for suspected neuroendocrine tumor.

Pathology showed calcitonin-positive neuroendocrine carcinoma of right arytenoid with 7 positive cervical lymph nodes (5/5 positive right level IIA, 1/3 positive right level III, and 1/5 positive left level IV). A 4 mm calcitonin-positive deposit of neuroendocrine carcinoma was present in right upper pole of the thyroid with adjacent intravascular tumor consistent with thyroidal metastasis from a primary laryngeal NET (moderately differentiated neuroendocrine tumor). Initial pathology did not report C-cell hyperplasia. However, on re-review of the images, it was felt that the calcitonin stain was less than ideal. Repeat staining was conducted and it is believed that there may be bilateral C-cell hyperplasia in the thyroid in addition to the tumor focus. RET mutation testing has been requested on one of the large metastatic tumor deposits in the lymph nodes.

While serum calcitonin level remained elevated, it did significantly decrease to 35 pg/mL postoperatively. The patient subsequently underwent adjuvant radiation therapy to the operative site delivered by intensity-modulated radiation therapy (IMRT). At 2-month follow-up, serum calcitonin had increased to 55 pg/mL, but without palpable recurrence on examination. At 6-month follow-up, serum calcitonin level had increased to 320 pg/mL. CEA remained normal. The patient complained of development of multiple subcutaneous nodules on chest, back, and forearm. These nodules were palpable on exam but had no associated overlying skin changes. PET scan showed interval development of multiple FDG avid nodules in subcutaneous tissue corresponding to the palpable nodules. There was no evidence of recurrence in the neck. Fine needle aspiration of one of the subcutaneous nodules was consistent with metastatic neuroendocrine carcinoma (positive for both calcitonin and synaptophysin). The patient was discussed at a multidisciplinary tumor board and it was determined that his disease was incurable given his distant metastasis (M1 stage). He was offered palliative chemotherapy and radiation; however the patient elected for no further treatment and is currently on hospice care.

## 3. Discussion

Neuroendocrine tumors of the larynx are rare, with just over 500 cases recorded in the literature since initially described by Goldman et al. in 1969 [11]. MDNC is the most common of the neuroendocrine tumors of the larynx [3]. MDNC is the second most common primary laryngeal malignancy following only squamous cell carcinoma. This tumor occurs 2-3 times more commonly in men and usually in heavy smokers. The average age at presentation is 61. Clinically, patients present with hoarseness, dysphagia, throat pain, and/or a neck mass [2]. Over 90% arise in the supraglottic larynx, in vicinity of the aryepiglottic fold, arytenoid, or false vocal cord [2]. Most thyroid carcinomas, on the other hand, invade the subglottis or trachea, sparing the supraglottis [12].

Medullary carcinoma of the thyroid (MCT) is another rare tumor of neuroendocrine origin. It accounts for ~3–5% of all thyroid gland cancers [13]. MTC arises from the C-cells of the thyroid gland which secrete calcitonin. The majority of the cases are sporadic (75%), although approximately 25% of MTC is hereditary due to germline mutation of the RET protooncogene, as seen in multiple endocrine neoplasias (MEN) 2A and 2B [13]. Sporadic MTC most commonly occurs in the 4th and 6th decade of life [14] and is slightly more common in females [13]. The classic presentation is a palpable solitary thyroid nodule. As C-cells are predominately located in the superior portion of the thyroid gland, the majority of MTC localize to the upper third of a lobe [13, 14].

Histologically, MDNC of the larynx and MTC have overlapping features, including epithelioid to spindle cells with moderate amounts of pale eosinophilic cytoplasm, an architectural arrangement in cords, nests, and solid sheets, characteristic nuclei with stippled neuroendocrine-type chromatin, scattered mitoses, and prominent vascular network [4]. Separating MDNC from MTC may be challenging since both tumors also stain positively for synaptophysin, calcitonin, and CEA [2]. TTF-1 has been useful in that it is strongly and diffusely positive in MTC but usually negative or only focally weakly positive in MDNC [2, 4]. Serum CEA is almost universally elevated in MTC. However, it has not been reported to be elevated in MDNC [2, 15]. Serum calcitonin is also almost invariably elevated in MTC. While many neuroendocrine tumors have been reported to secrete calcitonin, including paragangliomas, pheochromocytomas, gastric carcinomas, small cell pulmonary tumors, VIPomas, insulinomas, and enteropancreatic endocrine tumors [16], only 3 prior reports of hypercalcitoninemia have been reported in MDNC of the larynx.

Sweeney et al. [5] reported the first case of a neuroendocrine tumor of the larynx metastatic to cervical lymph nodes with an elevated serum calcitonin in 1981. This was followed by two additional patients reported by Smets et al. [6] and Insabato et al. [7]. In all three, thyroidectomy failed to disclose a primary thyroid neoplasm.  Table 1 compares our patient with the previous three.

**Figure 1:**
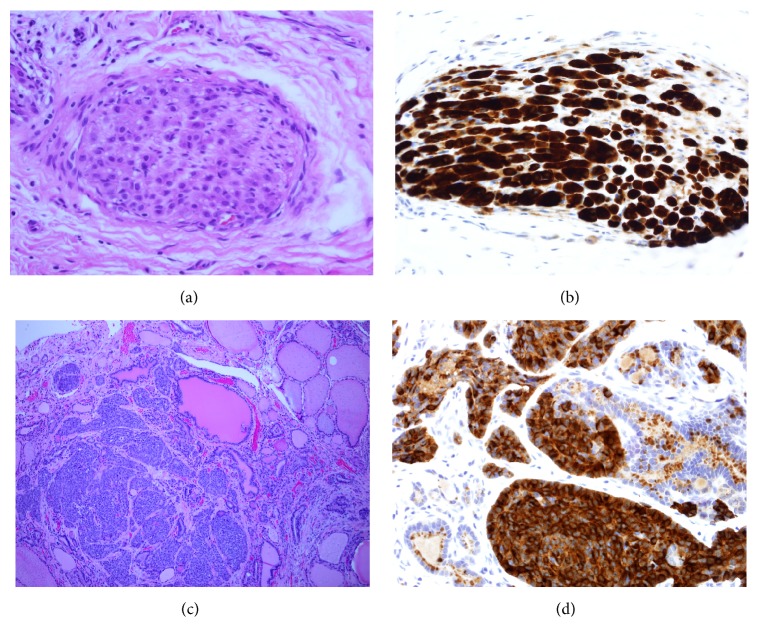
Tumor bed of laryngectomy and thyroid tumor, H&E stain and calcitonin immunostains. (a) H&E stain ×400: tumor bed, laryngectomy: solitary nerve twig infiltrated by plump epithelioid tumor cells. (b) Calcitonin immunostain ×400: tumor bed, laryngectomy: the infiltrating tumor cells are strongly immunoreactive for calcitonin. (c) H&E stain ×100: thyroid, right lobe nodule: 4 mm tumor nodule (left) adjacent to pink colloid-filled thyroid follicles (right). (d) Calcitonin immunostain ×400: thyroid: tumor cells are strongly reactive for calcitonin and can be seen focally invading into benign thyroid follicles.

**Figure 2:**
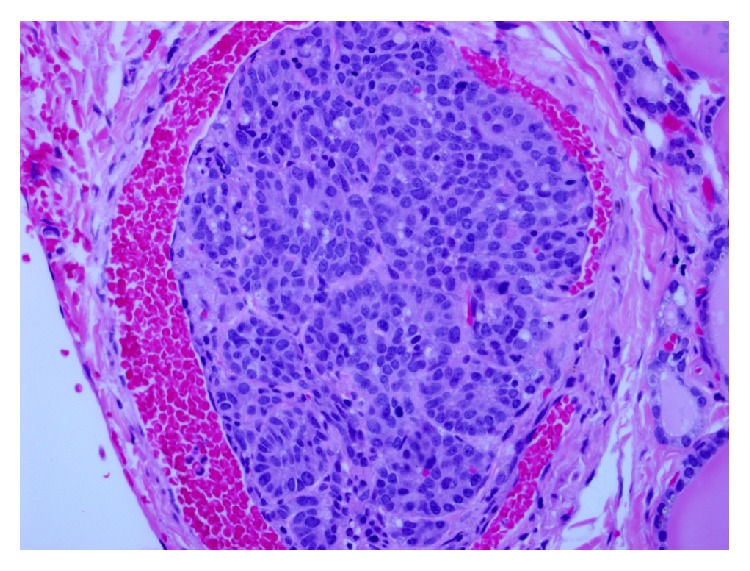
H&E stain ×400. Intravascular tumor adjacent to the right thyroid lobe nodule consistent with the diagnosis of intrathyroidal metastasis from a primary laryngeal neuroendocrine carcinoma.

**Figure 3:**
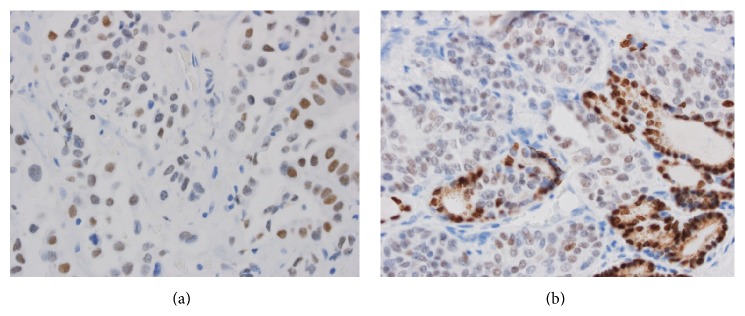
(a) Larynx, TTF-1 stain ×400: weak (light brown) nuclear staining of tumor cells. (b) Thyroid, TTF-1 stain ×400: weak (light brown) nuclear staining of tumor cells with strong (dark brown) nuclear staining of normal thyroid epithelium.

As in the previous three reports, the significantly elevated serum calcitonin level in our patient raised initial concerns for possible medullary thyroid carcinoma despite the supraglottic location of primary tumor. Additionally, in our patient, total thyroidectomy revealed 4 mm focus of tumor within the right lobe of the thyroid.  Figure 1 demonstrates H&E and calcitonin stains of tumor bed of laryngectomy and the 4 mm thyroid tumor. However, (1) the relatively small size of the thyroid tumor, (2) location of tumor in right arytenoid region (uncommon for MTC metastasis), (3) predominantly lateral cervical distribution of lymph node involvement (more typical for laryngeal than thyroid primary tumor), (4) extensive lymph-vascular space invasion by tumor present in cervical lymph nodes, and particularly (5) the presence of intravascular tumor adjacent to the right thyroid lobe nodule (Figure 2) were more consistent with the diagnosis of thyroidal metastasis from a primary laryngeal neuroendocrine carcinoma. MTC is associated with amyloid deposition in surrounding tissues. Congo red stains performed on both the thyroid tumor and one of the lymph node metastases were negative for demonstrable amyloid. The degree of calcitonin elevation in MTC correlates well with tumor volume [17]. Given our patient's tumor mass, a higher level of calcitonin would be expected for MTC. While more aggressive MTC may secrete less calcitonin, these tumors tend to have significantly elevated CEA levels [17]. Our patient's normal serum CEA level and focally positive TTF-1 stain are most consistent with MDNC. TTF-1 staining pattern in both the thyroid tumor and laryngeal tumor was similar to weak nuclear staining of tumor cells. This was in contrast to the strong nuclear staining of normal thyroid epithelium (Figure 3). To our knowledge, this is the first report of a laryngeal neuroendocrine tumor metastatic to the thyroid. We must acknowledge that the bilateral C-cell hyperplasia raises the possibility that the metastasis is actually a micro-MTC. RET testing is currently pending. But, autopsy studies have shown that a substantial proportion (up to 33%) of the normal adult population could have C-cell hyperplasia [18, 19].

Cutaneous metastatic carcinoma is a rare clinical finding. The overall incidence of cutaneous metastases for all types of carcinomas has been estimated to be 5.3% [20]. Skin metastasis of MTC is very rare, with only 16 cases reported in English literature to date [21, 22]. These metastases usually present as flesh-colored nodules that are tender and most commonly located on the scalp. However, laryngeal neuroendocrine tumors are known to metastasize to the skin and subcutaneous tissue. In a review by Woodruff and Senie of 127 published cases of laryngeal MDNC, 22% had metastasis to the skin or subcutaneous sites [23]. Thus, the skin involvement is another factor favoring a diagnosis of MDNC of the larynx.  Table 2 recaps the features favoring and disfavoring the diagnosis of MDNC of the larynx with metastasis to the thyroid.

In conclusion, the differential diagnosis in a patient with head/neck cancer and hypercalcitoninemia must include not only medullary thyroid cancer, but neuroendocrine tumors as well. Due to significant overlap in features, even pathological diagnosis may be difficult. Serum CEA levels and staining pattern for TTF-1 may be useful in distinguishing these two tumor types. While skin metastases are rare, this complication is more likely to occur in MDNC than MTC.

## Figures and Tables

**Table 1:** Comparison of reported MDNC of larynx with hypercalcitoninemia.

	Sweeney et al. (1981) [6]	Smets et al. (1990) [5]	Insabato et al. (1993) [7]	LaBryer et al. (present study)
Age/sex	54-year-old man	55-year-old man	69-year-old man	57-year-old man

Symptomatology	Hoarseness	Hoarseness and dysphagia	Hoarseness	Hoarseness, otalgia, odynophagia

Location of tumor	Left arytenoid, 3 cervical lymph nodes	Epiglottis, 3 submandibular lymph nodes	Right arytenoid, 1 cervical lymph node	Right arytenoid, 7 cervical lymph nodes

Immunostaining	Calcitonin+ CEA+ TTF-1 – no report	Calcitonin+ CEA+ TTF-1 – no report Cytokeratin+ Chromogranin A+ NSE+	Calcitonin+ CEA – no report TTF-1 – no report Cytokeratin+ Chromogranin A+ NSE+	Calcitonin+ CEA+ TTF-1+ (focally) Cytokeratin+ Chromogranin A+ NSE – not done

Serum calcitonin	1200 ng/L (ref < 200)	3790 pg/L (ref < 100)	970 pg/mL (ref < 300)	157 pg/mL (ref < 8)

Thyroidectomy specimen	Negative for MTC	Negative for MTC	Diffuse goiter, negative for MTC	4 mm focus of tumor with adjacent intravascular tumor

CEA = carcinoembryonic antigen; TTF-1 = thyroid transcription factor-1; NSE = neuron specific enolase; MTC = medullary thyroid carcinoma.

**Table 2:** Features favoring diagnosis of MDNC of larynx.

Features favoring diagnosis of MDNC of larynx	Features disfavoring MDNC of larynx
Age & sex	Bilateral C-cell hyperplasia of thyroid^*∗*^
Smoking history	
Clinical presentation of neck mass, hoarseness, and odynophagia	
Supraglottic location of primary tumor	
Normal serum CEA	
Serum calcitonin level compared to tumor volume	
Bilateral lateral cervical lymph node involvement	
Extensive lymph-vascular space invasion by tumor in lymph nodes	
Only focally positive TTF-1 staining in primary tumor and thyroid tumor	
Subcutaneous nodule metastases without overlying skin changes	
Negative amyloid stains of thyroid tumor and lymph node metastasis	

^*∗*^RET mutation testing pending.
